# Pre-PCI versus immediate post-PCI Impella initiation in acute myocardial infarction complicated by cardiogenic shock

**DOI:** 10.1371/journal.pone.0235762

**Published:** 2020-07-20

**Authors:** Veemal V. Hemradj, Mina Karami, Krischan D. Sjauw, Annemarie E. Engström, Dagmar M. Ouweneel, Justin de Brabander, Marije M. Vis, Joanna J. Wykrzykowska, Marcel A. Beijk, Karel T. Koch, Jan Baan, Robbert J. de Winter, Jan J. Piek, Antoine H. G. Driessen, Wim K. Lagrand, Alexander P. J. Vlaar, Jan Paul Ottervanger, José P. S. Henriques

**Affiliations:** 1 Department of Cardiology, Isala Hospital, Zwolle, The Netherlands; 2 Department of Cardiology, Amsterdam University Medical Center, Amsterdam, The Netherlands; 3 Department of Cardiology, Medisch Centrum Leeuwarden, Leeuwarden, The Netherlands; 4 Department of Cardiology, Erasmus Medical Center, Rotterdam, The Netherlands; 5 Department of Thoracic surgery, Amsterdam University Medical Center, Amsterdam, The Netherlands; 6 Department of Intensive Care Medicine, Amsterdam University Medical Center, Amsterdam, The Netherlands; Hospital Clinico San Carlos, SPAIN

## Abstract

**Background:**

In selected patients with an acute myocardial infarction (AMI) complicated by Cardiogenic shock (CS), mechanical circulatory support with Impella may be beneficial, although conclusive evidence is still lacking. Nevertheless, it has been suggested that Impella initiation prior to primary PCI might improve survival.

**Objective:**

To investigate the effect pre-PCI versus immediate post-PCI Impella initiation on short term mortality.

**Methods:**

A prospective, single center, observational study, was performed including all patients with STEMI complicated by CS, treated with primary PCI and Impella. Thirty day mortality was compared between patients with Impella initiation pre-PCI and immediately post-PCI.

**Results:**

A total of 88 patients were included. In the pre-PCI group (n = 21), admission heart rate was lower (84 versus 94 bpm, p = 0.04) and no IABP was implanted before Impella initiation, versus 17.9% in post-PCI group (n = 67), p = 0.04. Total 30-day all-cause mortality was 58%, and was lower in pre-PCI group, 47.6% versus 61.2% in the post-PCI group, however not statistically significant (HR 0.7, 95% CI 0.3–1.3, p = 0.21). Thirty-day cardiac mortality was significantly lower in the pre-PCI group, 19% versus 44.7% in the post-PCI group (HR 0.3, 95% CI 0.09–0.96, p = 0.042).

**Conclusion:**

Pre-PCI Impella initiation in AMICS patients was not associated with a statistically significant difference in 30-day all-cause mortality, compared to post-PCI Impella initiation.

## Introduction

Cardiogenic shock (CS) occurs in 5–10% of patients with acute myocardial infarction (AMI) and is still associated with 30-day mortality of approximately 40–60% despite emergent revascularization of the culprit lesion [1–4]. Medical treatment with inotropes and vasopressors do not improve survival. Current European and American guidelines give a class IIb recommendation for short-term mechanical circulatory support (MCS) in patients with refractory shock [5, 6]. However, routine MCS with an Intra-aortic balloon pump (IABP) initiated either before or after primary percutaneous coronary intervention (PCI) does not reduce mortality [7, 8]. Given the persistently high mortality in patients with AMI complicated by CS (AMICS), there is an urgent need to improve treatment in these patients. The use percutaneous MCS devices that provide more hemodynamic support than an IABP, such as an Impella, might be a possible beneficial strategy [9].

The Impella (Abiomed, Danvers, MA), is a catheter-based microaxial flow pump placed across the aortic valve into the left ventricle (LV). It unloads the LV and increases the cardiac output and coronary blood flow [10, 11]. It also increases the Cardiac Power Output, which has previously been shown to be the strongest hemodynamic predictor of mortality in AMICS patients [12, 13]. The Impella devices for LV-support, consist of the Impella 2.5, CP and 5.0. The Impella 2.5 and CP can be inserted percutaneously and provide a maximum output of respectively 2.5 L/min and 3.7 L/minute, whereas the Impella 5.0 needs to be inserted surgically and generates a maximum output of 5 L/min. Despite increased hemodynamic support with Impella, compared to IABP, there is conflicting evidence regarding the effect of Impella on clinical outcome [14–19]. However, in the studies with neutral or negative effect of Impella, most patients received Impella after primary PCI, while it has been suggested that Impella initiation prior to primary PCI might be a better strategy to improve survival than Impella initiation after primary PCI [20–22]. Unfortunately, previous studies assessing early Impella initiation in AMICS patients had major limitations, including a long or unclear period between Impella initiation and primary PCI and a small sample size.

Therefore, it remains unknown whether early Impella initiation may improve survival in AMICS patients as compared to delayed initiation. We investigated the effect of Impella initiation immediately before versus immediately after primary PCI on 30-day mortality in a large prospective cohort of AMICS patients.

## Methods

### Patient population

Data for this study are from the prospective cohort of patients who received an Impella at the Amsterdam University Medical Center (AUMC), The Netherlands. Our center is a high volume tertiary referral hospital with on-site cardiac surgery. All patient hospitalized between June 2006 and December 2016 with STEMI complicated by CS, treated with primary PCI and MCS with percutaneous Impella 2.5 or CP or surgical Impella 5.0 (Abiomed Inc., Massachusetts), were included in this study. Patients who did not receive Impella support in the same session as the primary PCI procedure, were excluded in order to avoid bias from delayed Impella therapy.

The Amsterdam University Medical Center Institutional Review Board approved the study and waived the requirement for informed consent in accordance with the Declaration of Helsinki. All data were fully anonymized.

### Treatment

All AMICS patients were treated with primary PCI according to standard PCI protocol. Before primary PCI, all patients received intravenous aspirin (500mg) loading dose, a P2Y12 blocker (either 600 mg of Clopidogrel or 180 mg of Ticagrelor) loading dose orally or via an esophageal tube when intubated and intravenous unfractionated heparin (5000 IU). Adjunctive treatment with glycoprotein IIb/IIIa inhibitors was at the discretion of the treating interventional cardiologist. Also the timing of Impella support (before or immediately after primary PCI) was left at the discretion of the treating interventional cardiologist. Post-PCI all patients received lifelong aspirin 100mg once daily and a P2Y12 blocker for 12 months (either 75 mg of Clopidogrel once daily or 180 mg of Ticagrelor twice daily).

Before 2012, only the Impella 2.5 and Impella 5.0 were available. After its introduction in 2012, the Impella CP became the first choice device in AMICS patients. Impella performance was set to a maximum level without console alarms (suction or position). Duration of Impella support was at discretion of the treating physicians. Upgrade to an Impella device with more hemodynamic support was considered when the device used was deemed to provide insufficient support. This was the case in patients who exhibited a combination of worsening hemodynamics and/or increased need for inotropes and vasopressors despite high Impella performance, together with an overall assessment of the patient and his/her neurological status. During Impella support, all patients were treated with unfractionated heparin in order to maintain an activated partial thromboplastin time (APTT) between 45 and 60 seconds.

Weaning was not formally protocolized but was evaluated daily by the treating physician and typically started on signs of hemodynamic recovery, usually 12–24 hours after PCI, when inotropes and vasopressors were reduced. Weaning usually occurred in two steps: from maximum possible support (P7–8) to approximately half support (P4–5) (if necessary patients were observed for several hours, typically overnight), to low-level Impella support (P2–3) before device removal. Device removal was typically also two-staged. First the device was pulled back from the left ventricle into the descending aorta. The device was not switched off, but set to level P1 in order to prevent thrombus formation. After 45–60 minutes of heparin cessation depending of last activated clotting time (ACT) level, the device was removed, followed by approximately 30 minutes of femoral compression.

### Definitions

CS was defined as a systolic blood pressure ≤ 90 mmHg for at least 30 minutes or the need for vasopressors to maintain a systolic blood pressure > 90 mmHg, clinical signs of pulmonary congestion and end-organ hypoperfusion (cool extremities, altered mental status, or a urine output of <30 ml/hour [4]. Patients who received Impella support immediately before initiation of the primary PCI (wiring of the infarct related artery), were defined as the pre-PCI group. Patients who received Impella support during or immediately after initiation of the primary PCI in the same procedure, were defined as the post-PCI group. Thirty day all-cause mortality was defined as mortality within the hospital admission or up to 30-days after the primary PCI (index procedure), whichever was longer. Cardiac mortality was defined as mortality due to refractory cardiogenic shock. Neurological mortality was defined as mortality due to irreversible post-anoxic brain injury leading to persisting coma, judged by a consulting neurologist and a concurring computed tomography scan.

### Analysis

Normally distributed continuous variables were reported as mean ± standard deviation (SD) and compared with ANOVA corrected for multiple testing by Bonferroni. Skewed distributed variables are presented as median and compared with the Wilcoxon rank sum test. Categorical variables are presented as proportions and compared with Chi-square test.

Age was dichotomized above and below the age of 75 years. Creatinine was dichotomized with the use of the clinical threshold for impaired renal function (>95 μmol/L for women and >110 for men). Hemoglobin was dichotomized using the clinical threshold for anemia (7.5 mmol/L for women and 8.5 mmol/L for man). Arterial pH, lactate, glucose and peak creatine-kinase (CK) MB were dichotomized according to the median value. Blood pressure at the time of Impella placement was dichotomized using the clinical threshold of 90 mmHg for systolic blood pressure and mean arterial pressure (MAP) of 60 mmHg. Cardiac arrest was categorized in: no cardiac arrest, return-off-spontaneous circulation (ROSC) time below 20 minutes and above 20 minutes.

Kaplan Meier analyses were calculated and a log-rank test was used to compare the clinical outcomes between groups.

A Cox proportional-hazards regression model was used to calculate both uni-variable and multi-variable adjusted hazard ratios, with calculation of 95% confidence intervals. Not all univariate significant variables were added to the model because of the limited number of patients and consequent loss of power of the model. A covariate was removed from the model if its significance level exceeded p = 0.10. Variables included in the multivariable analysis were age, sex, admission heart rate and IABP placement before Impella initiation. Analyses were performed with SPSS (version 26.0, Chicago, Illinois).

## Results

### Patient population

A total of 88 patients were included in this study, with 21 patients (23.9%) in the pre-PCI and 67 patients (76.1%) in the post-PCI group. Baseline characteristics are summarized in Table 1. Patients were 60 ± 10 years old and 82% was male. A total of 82% of patients suffered an anterior located myocardial infarction, 69% had multivessel disease and 60% experienced cardiac arrest before Impella initiation. Angiographic success was achieved in 76% of the patients. The majority of patients were treated with catecholamines and/or inotropes and were mechanically ventilated.

**Table 1:** Baseline characteristics of AMICS patients treated with primary PCI and Impella.

		Pre-PCI	Post-PCI	
		(n = 21)	(n = 67)	*p*
**Clinical characteristics and risk factors**				
Age (years)		60.0 ± 11.7	61.2 ± 10.0	0.63
Male sex, n (%)		18 (85.7)	54 (80.6)	0.75
Body mass index (kg/m2)		25.7 [23.6–27.4]	26.0 [24.5–27.6]	0.69
Cardiovascular risk factors, n (%)				
	Current smoking	8 (40.0)	23 (44.2)	0.79
	Hypertension	8 (40.0)	23 (35.9)	0.79
	Diabetes mellitus	5 (25.0)	9 (13.4)	0.29
Prior myocardial infarction, n (%)		3 (16.7)	13 (19.7)	1.00
Prior TIA or stroke, n (%)		1 (5.0)	1 (1.5)	0.41
Known peripheral arterial disease, n (%)		1 (5.6)	4 (6.3)	1.00
Prior PCI or CABG, n (%)		5 (23.8)	10 (15.4)	0.50
**Clinical characteristics on admission to the cardiac catheterization laboratory**				
Blood pressure values				
	Mean arterial pressure	59 [48–66]	67 [56–77]	0.51
	Systolic blood pressure (mmHg)	80 [67–95]	86 [71–101]	0.55
	Diastolic blood pressure (mmHg)	49 [40–58]	58 [45–67]	0.31
	Heart rate (beats per minute)	84 [61–108]	94 [80–112]	0.04
Catecholamines or inotropes, n (%)		19 (90.5)	59 (88.1)	1.00
Mechanical ventilation, n (%)		18 (85.7)	60 (89.6)	0.69
Cardiac arrest, n (%)		11 (52.4)	42 (62.7)	0.62
	Out of hospital cardiac arrest, n (%)	10 (47.6)	29 (43.2)	0.17
	Witnessed arrest, n (%)	10 (47.6)	36 (53.7)	0.29
	First rhythm VT/VF/AED, n (%)	8 (38.1)	35 (52.2)	0.23
	Time till return of spontaneous circulation (min)	25 [18–50]	20 [11–53]	0.88
Traumatic injuries at admission, n/n (%)		2 (9.5)	4 (6.0)	0.62
Laboratory values on admission				
	Lactate (mmol/L)	7.7 [4.2–9.6]	6.5 [3.8–10.4]	0.94
	Hemoglobin (mmol/L)	8.4 [7.4–9.0]	8.7 [7.6–9.4]	0.37
	Creatinine (umol/l)	111 [98–133]	112 [89–128]	0.59
	Glucose (mmol/L)	13.8 [11.0–18.0]	15.7 [11.1–20.4]	0.67
	Arterial pH	7.22 [6.94–7.34]	7.18 [7.05–7.28]	0.70
**Primary percutaneous coronary intervention**				
Ischemic time (min)		198 [118–414]	150 [96–219]	0.14
Infarct-related artery, n (%)				0.29
	Left main	7 (33.3)	18 (26.9)	
	Left anterior descending	10 (47.6)	37 (55.2)	
	Left circumflex	1 (4.8)	9 (13.4)	
	Right coronary artery	3 (14.3)	3 (4.5)	
Multi-vessel disease, n (%)		17 (81.0)	44 (65.7)	0.27
TIMI flow pre-PCI, n (%)				0.51
0		13 (61.9)	52 (77.6)	
1		2 (9.5)	3 (4.5)	
2		2 (9.5)	5 (7.5)	
3		4 (19.0)	7 (10.4)	
TIMI flow post-PCI, n (%)				0.38
0		0 (0)	1 (1.5)	
1		2 (9.5)	2 (3.0)	
2		2 (9.5)	14 (20.9)	
3		17 (81.0)	50 (74.6)	
Intra-aortic balloon pump before Impella placement, n (%)*		0 (0)	12 (17.9)	0.03

Data are displayed as percentile (frequency), mean ± standard deviation or median [25th percentile - 75th percentile]. VF: ventricular fibrillation; VT: ventricular tachycardia; PCI: percutaneous coronary intervention; CABG: coronary artery bypass grafting

* 6 patients received IABP prior to primary PCI and 6 patients after primary PCI but before Impella.

Patients in the pre-PCI group had a lower heart rate at admission (84 bpm versus 94 bpm, p = 0.04). IABP prior to Impella initiation was statistically different between the pre-PCI and post-PCI group, respectively 0 versus 12 patients (0% versus 17.9%, p = 0.04). Six patients in the post-PCI group received IABP prior to primary PCI and prior to Impella initiation and 6 patients received IABP after primary PCI but prior to Impella initiation. Other baseline characteristics were not statistically different.

### Clinical course

Characteristics of the clinical course are summarized in Table 2. The initial Impella strategy consisted of Impella 2.5 in 35 patients (39.8%), Impella CP in 45 patients (51.1%) and Impella 5.0 in 8 patients (9.1%) and was not different between the pre- and post-PCI group. Eleven patients (12.5%), were upgraded to a higher flow support device (Impella 5.0 or VA-ECMO), not different between the pre-and post-PCI group. Median Impella support duration in survivors was 84 hours in the pre-PCI group and 75 hours in the post-PCI group (p = 0.12). Admission to the ICU was not statistically different between the pre- and post-PCI group (85% versus 89.4%, p = 0.59). In the pre-PCI group, there was a trend towards less mechanical ventilation (85.7% versus 97.0%, p = 0.051) and shorter hospitalization on the ICU (median of 8 versus 13 days, p = 0.16) in survivors.

**Table 2:** Clinical course of AMICS patients treated with primary PCI and Impella.

	Pre-PCI	Post-PCI	
	(n = 21)	(n = 67)	*P*
**Mechanical circulatory support**			
Duration of Impella support			
Duration of Impella support in survivors, (median, hours) *	84 [33–162]	75 [50–125]	0.84
Duration of Impella support in non-survivors, (median, hours) *	62 [15–230]	31 [6–62]	0.18
First Impella Device			0.87
Impella 2.5	8 (38.1)	27 (40.3)	
Impella CP	11 (52.4)	34 (52.2)	
Impella 5.0	2 (9.5)	6 (9.0)	
Change of mechanical support device, n (%)	3 (14.3)	8 (11.9)	0.77
Upgrade to Impella 5.0	3 (14.3)	6 (9.0)	
Upgrade to ECLS	-	2 (3.0)	
Device failure requiring extraction of the device, n (%)	1 (4.8)	-	0.07
**During admission**			
Inotropic or vasopressor therapy, n (%)	19 (90.5)	63 (94.0)	0.57
Renal replacement therapy, n (%)	9 (42.9)	26 (38.8)	0.74
Mechanical ventilation, n (%)	18 (85.7)	65 (97.0)	0.05
Peak CKMB, μmol/L	445 [141–1023]	454 [204–804]	0.28
Blood products, n (%)	13 (61.9)	38 (56.7)	0.67
Admission to the intensive care unit, n (%)	17 (85.0)	59 (89.4)	0.59
Days on the intensive care unit in survivors	8 [2–17.7]	13 [4–31]	0.16
Days on the intensive care unit in non-survivors	3 [0.5–10.5]	2.5 [1–4]	0.95

* Sum of support duration of all given support devices. PCI = percutaneous coronary intervention. ECLS = extra corporeal life support.

### Complications

There were no significant differences in the frequency of complications between the two groups (Table 3). Two patients were diagnosed with stroke during admission (2.3%), both in the post-PCI group. Device related vascular complications occurred in 14 patients (15.9%) and occurred more often in the pre-PCI group (23.8% versus 13.4%, p = 0.25). Two patients (9.5%) in the pre-PCI group had a major access site related bleeding versus 7 (10.4%) in the post-PCI group. Four patients experienced limb ischemia requiring surgery (2 in both groups). Clinically relevant hemolysis occurred in 9.1% of the patients, all in the post-PCI group. Non-device related bleeding occurred in 13 patients (14.8%).

**Table 3:** Clinical outcome of AMICS patients treated with primary PCI and Impella.

			Pre-PCI	Post-PCI	
			(n = 21)	(n = 67)	*p*
**In-hospital outcome**					
Stroke, n (%)			-	2 (3.0)	0.42
	Hemorrhagic stroke		-	1 (1.5)	
	Ischemic stroke		-	1 (1.5)	
Device related vascular complication, n (%)			5 (23.8)	9 (13.4)	0.25
	Limb ischemia		2 (9.5)	2 (3.0)	
	Access site related bleeding		3 (14.3)	7 (10.4)	
		Major bleeding	2 (9.5)	7 (10.4)	
		Minor bleeding	1 (4.8)	-	
	Access site infection				
Non-device related bleeding			3 (14.3)	10 (14.9)	0.94
		Gastro-intestinal bleeding	1 (4.8)	5 (7.5)	
		Other location	2 (9.5)	5 (7.5)	
Clinically relevant hemolysis, n (%)			-	8 (11.9)	
**Follow-up outcome**					
	30-day all-cause mortality, n (%)		10 (47.6)	41 (61.2)	0.27
	Cardiac mortality		4 (19.0)	30 (44.7)	0.03
	Neurological mortality		3 (14.2)	7 (10.4)	0.69
	Other reason		3 (14.2)*	4 (6.0)**	0.20
	1 year all-cause mortality, n (%)		11 (52.4)	43 (64.2)	0.33

* persisting respiratory insufficiency due to aspiration pneumonia in 1 patient and recurrent heart failure in 2 patients

** haemorrhagic shock due to device related retroperitoneal bleeding in 1 patient, in-hospital cardiac arrest due to obstructive shock/ lung embolism in 1 patient, sepsis and progressive metastases of underlying colon carcinoma in 1 patient and recurrent heart failure in 1 patient.

### Outcome

**Fig 1:**
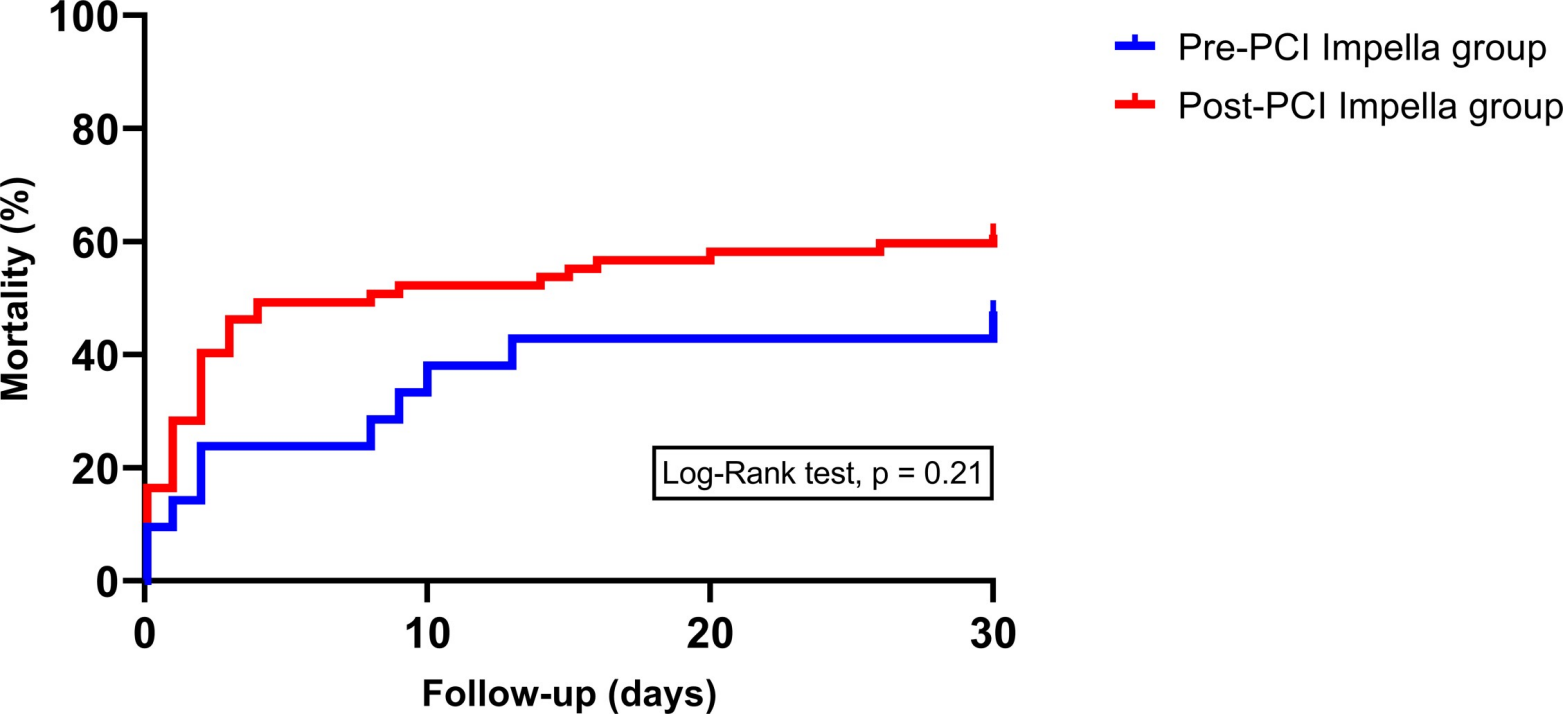
Short term all-cause mortality in AMICS patients treated with primary PCI and pre- versus post-PCI Impella support.

**Fig 2:**
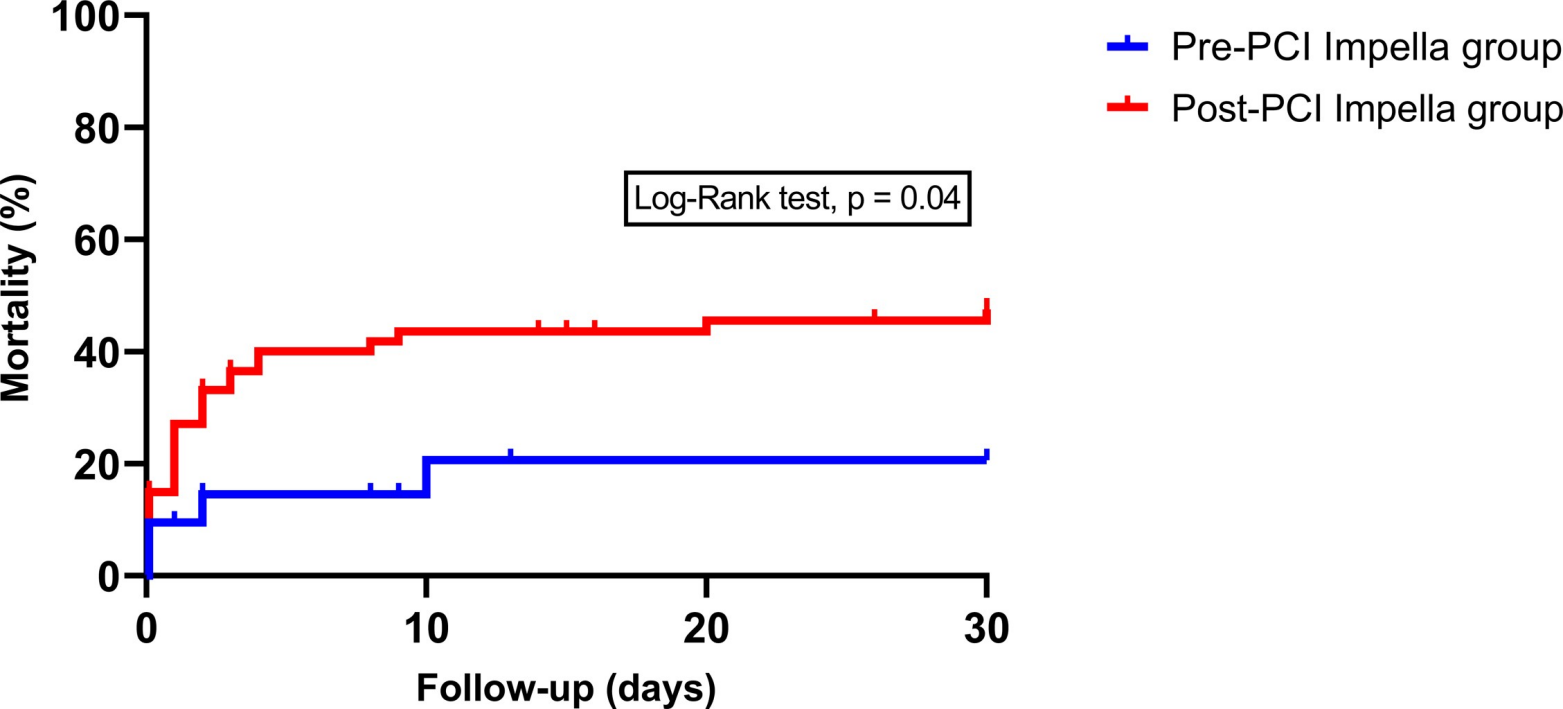
Short term cardiac mortality in AMICS patients treated with primary PCI and pre- versus post-PCI Impella support.

The overall all-cause 30-day mortality was 58.0%, with 47.6% in the pre-PCI group versus 61.2% in the post-PCI group (HR 0.7, 95% CI 0.3–1.3, p = 0.21), Fig 1. The 30-day cardiac mortality was significantly reduced in the pre-PCI group, 19.0% versus 44.7% in the post-PCI group (HR 0.3, 95% CI 0.09–0.96, p = 0.04), Fig 2. Neurological mortality did not differ significantly between the pre-PCI and post-PCI group (respectively 14.2% versus 10.4%, HR 1.4, 95% CI 0.3–6.1). After multivariable analysis, there was also no statistically significant difference in 30-day all-cause mortality between the pre-PCI and post-PCI group (HR 0.8, 95% CI 0.4–1.6, p = 0.50).

## Discussion

This prospective single-center observational study in AMICS patients evaluated the potential association between timing of Impella initiation and mortality. The key findings of our study were that 1) 30-day all-cause mortality was still very high (58%) despite successful primary PCI and mechanical circulatory support with Impella, 2) pre-PCI Impella initiation was not associated with a statistically significant difference in 30-day all-cause mortality, compared to post-PCI Impella initiation, 3) pre-PCI Impella initiation was associated with a clinically and statistically significant reduction in 30-day cardiac mortality compared to post-PCI Impella initiation.

This study included a very high risk AMICS patient population with nearly 50% of patients admitted after an out-of-hospital cardiac arrest, which in itself is associated with poor survival [23, 24]. This study population was similar to populations in previous trials that assessed early Impella initiation in AMICS patients [20–22, 25]. In these studies a significant mortality reduction was reported in patients who received Impella pre-PCI. Basir et al. performed a retrospective study in 287 patients [20]. Patient data was retrieved from the cVAD registry. However, in their analysis, patients who received Impella till 24 hours post-PCI were also included, which could have led to bias due to more profound CS in the group of patients with late Impella implantation. In the present study only patients who received Impella in the admission procedure, either pre- or post-PCI, were included. Moreover, important potential confounders that differed significantly at baseline (i.e. unsuccessful PCI) were not included in their multivariable model and the exact number of patients who had Impella initiation before PCI was not stated. Jensen et al. and Loehn et al. also reported a beneficial effect of early Impella initiation, although both studies had a small sample size [21, 22]. In the study by Loehn et al, all patients were treated with the Impella CP, which provides more hemodynamic support than the Impella 2.5. In our study, 40% of patients were treated with the Impella 2.5, which was equal between the pre-PCI and post-PCI group.

Neurological mortality did not differ significantly between the pre- and post-PCI group. This finding stresses out the necessity to be able to accurately predict which patients will recover from coma in the acute phase and which patients will not. Part of admitted patients after out-of-hospital cardiac arrest will not recover from coma, no matter the effort. Probably, (early) restoration of hemodynamics will not be beneficial in this subgroup of patients. However, till date it is impossible to accurately predict which patients will recover from coma after AMICS or cardiac arrest and which patients will not.

Interestingly, in our analysis, cardiac mortality was significantly lower in the pre-PCI group. Till date, no clinical studies with hemodynamic support devices have extended the benefit of primary PCI in AMI or AMICS yet. Especially in CS it is postulated that stabilizing the systemic circulation with a hemodynamic support device could reduce mortality, as longer duration of CS, with subsequently the occurrence of multi-organ failure and systemic inflammatory response syndrome (SIRS) is associated with higher mortality [26, 27]. Early implantation of a hemodynamic support device, i.e. pre-PCI, could be of importance, since multiple pre-clinical studies showed a beneficial effect on infarct size by unloading the left ventricle and lowering myocardial oxygen demand [28–30]. The question remains however, whether the possible benefits of pre-PCI hemodynamic support, i.e. with a direct LV unloading device as Impella, justify a longer time to restoration of flow in the culprit artery [31]. In a recent clinical pilot study (n = 50) by Kapur et al, it was shown for the first time that pre-PCI initiation of Impella CP was not associated with larger infarct size despite an average of 30 minutes longer ischemic time [32]. However, reduction of infarct size with a pre-PCI strategy, still needs to be confirmed in the setting of AMICS.

While our study showed a significantly reduced 30 day cardiac mortality in the pre-PCI group, the 30 day all-cause mortality did not differ significantly. Our findings stress out the need for confirming data from an adequately powered RCT. The results of the DanGer Shock trial, which randomizes AMICS patients between Impella CP prior to PCI and current guideline driven therapy, are therefore eagerly awaited [33].

### Limitations

There are several limitations that apply to this analysis. This analysis is a single center study with an observational design. The sample size is relatively small. Moreover, timing of Impella initiation was not dictated per protocol which may have led to selection bias. It remained unclear what the reason for pre-PCI Impella initiation was. Therefore, potential unmeasured confounding bias cannot be ruled out. We performed multivariable analysis, but this was limited due to the small sample size of our cohort. Finally, there are several factors that might have influenced the results, such as experience with the device, change of therapy over time, improvement of general therapy of AMICS patients over time, and change in patient selection over time. Given the abovementioned limitations, the findings of our study should be interpreted as hypothesis generating.

## Conclusion

Despite early revascularization and Impella support, 30-day mortality was still very high in patients with AMICS. Pre-PCI Impella initiation in AMICS patients was not associated with a statistically significant difference in 30-day all-cause mortality, compared to post-PCI Impella initiation. Future large randomized clinical trials will be needed to assess whether pre- or post- PCI Impella initiation increases survival in these patients with a priori high mortality.

## Supporting information

S1 Table Definitions. (DOCX)

S1 Dataset (XLSX)
