# Supplementary material for: Pre-PCI versus immediate post-PCI Impella initiation in acute myocardial infarction complicated by cardiogenic shock
Source: PLoS One. 2020 Jul 20;15(7):e0235762. doi: 10.1371/journal.pone.0235762 (PMC7371192; doi:10.1371/journal.pone.0235762)
Supplement: S1 Table — (DOCX) [file pone.0235762.s001.docx]

**Table S1. Definitions**

| **Event** | **Definition** |
| --- | --- |
| Ischemic time | Time from symptom onset to first balloon deployment during PCI |
| Multivessel disease | One or more stenosis of more than 50% in a non-culprit vessel |
| Angiographic success | TIMI flow post PCI 3 |
| Device-related vascular complication | Limb ischemia requiring extraction of the device, an access site infection, or an access site-related bleeding |
| Access site-related bleeding was subdivided into major and minor bleeding. | - Major bleeding: a bleeding associated with a serum hemoglobin level decrease of 3.1 mmol/L (5 g/dL), a necessity of a minimum of two packed cells of blood product transfusion or the need for surgical intervention - Minor bleeding: access site bleedings that were reported on the patient’s chart or the hospital discharge letter, but did not fit the definition of a major bleed |
| Hemolysis | Clinically relevant hemolysis requiring extraction of the device or blood transfusion(1) |
| Hemorrhagic or ischemic stroke | Diagnosis confirmation by a neurologist and a concurring computed tomography scan |
| Renal insufficiency on admission | Clinical threshold of Creatinine >95 μmol/L for women and >110 μmol/L for men (2) |
| Anemia | Clinical threshold of Hemoglobin < 7.5 mmol/L for women and < 8.5 mmol/L for men (2) |

**References**

1. Ouweneel DM, Eriksen E, Sjauw KD, van Dongen IM, Hirsch A, Packer EJ, et al. Percutaneous Mechanical Circulatory Support Versus Intra-Aortic Balloon Pump in Cardiogenic Shock After Acute Myocardial Infarction. J Am Coll Cardiol. 2017;69(3):278-87.

2. Ouweneel DM, de Brabander J, Karami M, Sjauw KD, Engstrom AE, Vis MM, et al. Real-life use of left ventricular circulatory support with Impella in cardiogenic shock after acute myocardial infarction: 12 years AMC experience. Eur Heart J Acute Cardiovasc Care. 2018:2048872618805486.
